# Application of improved fuzzy best worst analytic hierarchy process on renewable energy

**DOI:** 10.7717/peerj-cs.453

**Published:** 2021-04-13

**Authors:** Priyanka Majumder, Valentina Emilia Balas, Arnab Paul, Dayarnab Baidya

**Affiliations:** 1 Department of Basic Science and Humanities, Techno College of Engineering Agartala, Agartala, Tripura, India; 2 Department of Automation and Applied Informatics, Aurel Vlaicu University of Arad, Arad, Romania; 3 Department of Electronics & Instrumentation Engineering, National Institute of Technology Silchar, Assam, Silchar, India

**Keywords:** Fuzzy BWM, Fuzzy AHP, Trapezoidal fuzzy number, Climate change, Hydro power plant

## Abstract

In this work, a novel fuzzy decision making technique namely trapezoidal fuzzy Best-Worst method (fuzzy BWM) is developed which is based on Best-Worst method (BWM) and Trapezoidal fuzzy number. The real motive behind our work is to take a broad view of the existing fuzzy BWM based on triangular fuzzy number by trapezoidal fuzzy number. Also, we have presented a new hybrid MCDM technique called as Trapezoidal fuzzy Best Worst Analytic Hierarchy based on proposed trapezoidal fuzzy BWM and existing trapezoidal fuzzy Analytic Hierarchy Process (AHP). BWM approach is employed in evaluating the PV of considering criteria and trapezoidal fuzzy AHP is used to assess the local priority vale (PV) of considering alternatives (or indicators) of a decision problem. Moreover it used to identify the most significant alternative which is responsible for performance efficiency of a hydro power plant under climatic scenario. From the result, it is undoubtedly found that hydraulic had is most responsible indicator. Further, the CR (consistency ratio) value which is determined by our proposed trapezoidal fuzzy BWM is less than that of existing BWM and fuzzy BWM techniques. Finally, we have validated our result by comparative study, scenario analysis and sensitivity analysis.

## Introduction

Decision-making suggests the mining of appropriate different from a collection of indicators (Plous, 1993). DM is termed as Multi-Criteria Decision-Making (MCDM) that is applied in several fields, like management, economics, and engineering (Stewart, 1992; Zavadskas & Turskis, 2011; Wallenius et al., 2008). Since 1960, more than a few MCDM Techniques have been developed, proposed and implemented successfully in many application areas. The MCDM ways conjointly utilized in the fuzzy surroundings as a result of indistinctness in human thinking, complexity, therefore the high aptitude of fuzzy data to replicate decision data (Guo & Zhao, 2017). Thus, researchers are paying attention to use fuzzy-based MCDM tools for example fuzzy Data Envelopment Analysis (DEA) (Rufuss et al., 2018; Jafarzadeh, Akbari & Abedin, 2018), fuzzy AHP (Rufuss et al., 2018; Nazari et al., 2018; Jeou-Shyan et al., 2011; Ahmed & Kilic, 2019), fuzzy VIKOR (VlseKriterijumska Optimizcija I Kaompromisno Resenje in Serbian) (Yager, 2017; Mandal et al., 2015; Tadić, Zečević & Krstić, 2014), fuzzy ELECTRE (Elimination and Choice Translating algorithm) (Tadić, Zečević & Krstić, 2014; Peng et al., 2015), fuzzy ANP (Analytic Network Process) (Tadić, Zečević & Krstić, 2014; Mikhailov & Singh, 2003) and fuzzy TOPSIS (Technique for Order Preference by Similarity to Ideal Solution) (Han & Trimi, 2018; Dwivedi, Srivastava & Srivastava, 2018; Chen & Hong, 2014).

A very well known MCDM method called as BWM was presented in 2015 (Rezaei, 2015). The importance of inconsistency assists the decision-makers (DMs) to experience if the last choices are complete properly or not, and also to find out how consistent the opinions are (Pohekar & Ramachandran, 2004). The easiness, correctness and fewer redundant make BWM more useful (Rezaei, 2015). Meanwhile in 2017, fuzzy-based MCDM methods known as fuzzy BWM have been proposed by Guo & Zhao (2017). Recently, various authors showed their interest on BWM method in fuzzy environment such as Ghoushchi, Yousefi & Khazaeili (2019), Maghsoodi et al. (2019) and Tian, Wang & Zhang (2018). Ahmed et al. (2017) utilized the fuzzy partitioning method in the Crone’s disease classification. Azar et al. (2013) used fuzzy linguistic hedges for the selection for differential diagnosis of Erythemato-Squamous diseases. Balas & Balas (2008) explained the usefulness of giving controllers with various amounts of world knowledge: general knowledge on system theory, specific information on the processes, etc.

A triangular or trapezoidal fuzzy number is typically focussed to provide the decision groups perception of indicators regarding every criteria. In fact, a triangular fuzzy number could be a special case of a trapezoidal fuzzy number (Zheng et al., 2012). Once the two important values are an equivalent number, the trapezoidal fuzzy number becomes a triangular fuzzy number (Zheng et al., 2012). From these literatures, it can be said that a trapezoidal fuzzy number is most suitable than triangular fuzzy number.

### Objective

It is known that a triangular fuzzy number can be a specific case of a trapezoidal fuzzy number (Zheng et al., 2012), thus the objective is to develop a novel MCDM technique based on trapezoidal fuzzy number and BWM named as trapezoidal fuzzy Best-West Method (TrFBWM).

The next aim is to suggest a hybrid MCDM method based on trapezoidal fuzzy number. In this aspect, our proposed study is based on two MCDM methods such as fuzzy BWM and fuzzy AHP. Here fuzzy BWM is used to find out the PV of criteria and fuzzy AHP is applied for evaluate the PV of each indicators. In the present investigation, BWM is used because it erases three drawbacks of AHP. Firstly, BWM requires less number of comparisons than that in AHP, because BWM develops the PV of criteria supporting the vectors of pairwise comparisons whereas the AHP utilizes the total matrix of comparisons (Mi et al., 2019). Secondly, in BWM, only integer’s scale is employed within the structured comparing process. In this regard, the difficulty of comparisons reduces once more. Additionally, the essential scores are abundant nearer to person views and knowledge, and this causes the assessment method to become much easier. The third advantage of BWM is that it has improved functioning in arguing the consistency of pairwise comparisons as the redundant comparisons are eliminated. This makes the outcomes by BWM more reliable than those developed by AHP. Also a triangular or trapezoidal fuzzy number is typically adopted to express the decision groups perception of indicators functioning with regard to every criterion. In fact, a triangular fuzzy number could be a special case of a trapezoidal fuzzy number (Zheng et al., 2012). Hence, a trapezoidal fuzzy number can deal with more general situations. Depending on the above literatures and the advantages of trapezoidal fuzzy number, it is adopted in our work which is another objective of our study.

Some studies (Spalding-Fecher et al., 2016; Carvajal et al., 2017; Majumder, Majumder & Saha, 2018; Majumder et al., 2019) on productivity of HPP under climatic vulnerabilities and considering sustainability factors were never tried which is why, this investigation can try and develop a decision support model wherever most important options can rate the performance of HPP supported global climate change. Moreover, the influence of indicators are enclosed within the decision making process as per their significance with regard to the climatic parameter and also the influence of climatic parameter are rated by their ability to impact the property factors which is able to be accustomed rank totally different power plants supported the climatic vulnerability.

### Novelty

The novelty of present study lies in developing a new trapezoidal based fuzzy BWM decision making technique. Second novelty is to propose a new hybrid MCDM technique, based on BWM and AHP using trapezoidal fuzzy number. Further, we tend to use trapezoidal fuzzy number in our proposed method to deal with more general solutions to examine the influence of climate change in our real life. Since, climatic change has emerged as the biggest challenge for the performance of hydropower plant (HPP), so the study of the performance hydropower plant with respect to climate change is another uniqueness of our study.

## Literature review

Making decision brings out the suitable alternative from a group of alternatives. It is done on the basis of various criteria, named as MCDM, appropriate to various fields, like engineering, economics, management, etc. (Stewart, 1992; Zavadskas & Turskis, 2011; Wallenius et al., 2008). The accustomed method of the MCDM strategies depends on the ranking of all the alternatives and selecting the superior one by an approach in the light of some criteria. There are a variety of MCDM techniques which have been used by scientists, economists, mathematicians to solve the real life problems. One of such technique is AHP which is used for frequent applications in multiple sectors of economics, politics, and engineering that was designed by Saaty (1980). Regardless of its widespread applicability, the AHP method has various limitations: firstly, it uses a big number of pairwise comparisons to come to a decision. This condition clogs its application to the most essential issues. Secondly, it has a lack of consistency. So, it is required to check the CR as the CR must be less than 1 for the method to be consistent.

The technique BWM is considered to prevail over the difficulties of AHP. Youssef (2020) proposed an approach that incorporates TOPSIS and the BWM for the ranking CSPs using evaluation criteria characterizing their services. Knowing of the fewer pairwise comparison and also the excessive consistency of the matrix of pairwise comparison within the BWM than that in AHP, the BWM will be as well-liked as AHP almost immediately following. Compared to AHP, BWM only performs reference comparisons, meaning that it only has to determine the priority of the best criterion over all the other criteria and the priority of all the criteria over the worst criterion. Thus BWM significantly increase the overall consistency of the problem compared to AHP. Moreover, this technique has some disadvantages such as vagueness in human opinions and uncertainty in the information related to the criteria (Zhao & Guo, 2015).

## Trapezoidal fuzzy best-worst analytic hierarchy process

**Figure 1:**
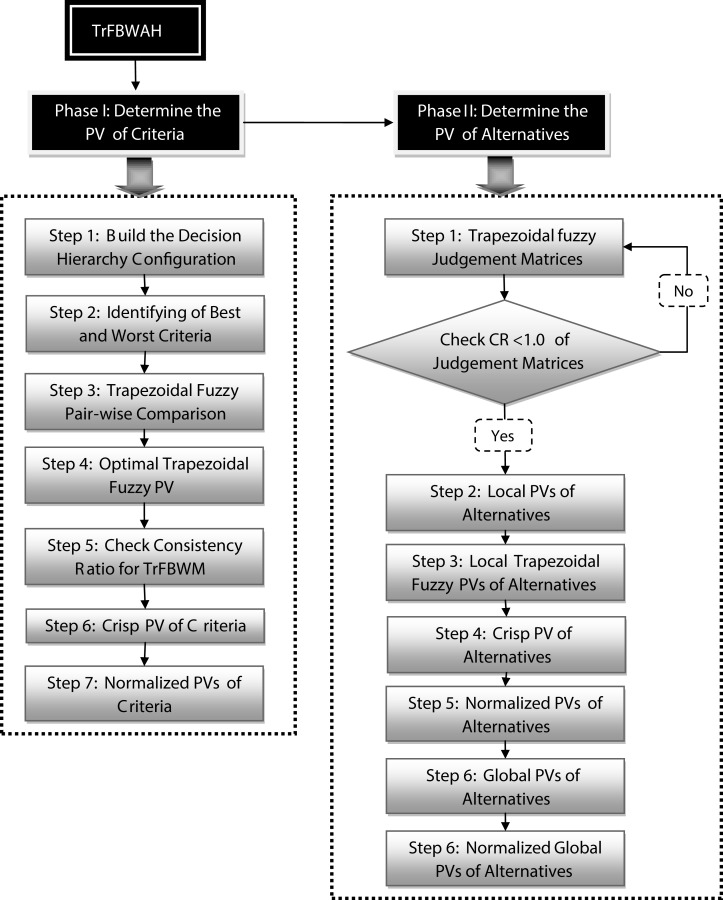
Total scenario of proposed method.

The proposed method namely Trapezoidal fuzzy Best Worst Analytic Hierarchy (TrFBWAHP) consists of two phases respectively phase I and phase II. Phase I is used to evaluate PV of criteria and Phase II is utilized to determine the PV of indicators. Figure 1 represents computational process of proposed method.

**Phase-1:** Best–Worst Method (BWM) is a novel method to evaluate the PV of criteria. In this study, a modified fuzzy BWM approach namely Trapezoidal Best-West Method (TrFBWM) to evaluate the PV of each criterion is proposed. The TrFBWM techniques consist of five steps. The evaluation steps are given in below:

**Step-1: Define decision making Criteria:** In MCDM techniques criteria are significant for selection of most important indicator. Let $C=\left\{c_{1},c_{2},...,c_{n}\right\}$ and $A=\left\{A_{1},A_{2},...,A_{p}\right\}$ denote the sets of criteria and indicator respectively. The performance of each indicator is depending on the set of elements of *C*.

So, $PV(A_{i})=f_{i}\left(c_{1},c_{2},...,c_{n}\right);\;\;\forall i=1,2,...,p$

**Step-2: Selection of Best and Worst Criteria:** Next step decision maker (DM) selects the best and worst criteria among the sets of criteria. DM may be selecting these best and worst criteria with help different survey like expert, literature, media survey etc. Let $c_{B}$ and $c_{w}$ be the best and worst criteria respectively. Then $c_{B},\;c_{w}\;\in \;C$.

**Step-3: Trapezoidal Fuzzy Pairwise Comparison:** Total Pairwise comparison matrix is not requiring. In Step-3 pairwise comparison are divided into two parts which are described in step 3.1 and 3.2. In Table 1, Trapezoidal fuzzy pairwise comparisons between criteria have done by using Linguistic measures of importance. Thus convert each Linguistic term into trapezoidal fuzzy measures of importance which is shown in below Table 1.

**Table 1:** Linguistic measures of importance used for pairwise comparisons.

Linguistic Term	Trapezoidal fuzzy measures of importance (l,m,n,s)	Score Index (SI)
Absolutely More Important (AMI)	$\left(8,\frac{17}{2},9,9\right)$	9
Very Strong Important (VSI)	$\left(6,\frac{13}{2},\frac{15}{2},8\right)$	7
Essentially Important (ESI)	$\left(4,\frac{9}{2},\frac{11}{2},6\right)$	5
Weakly Important (WI)	$\left(2,\frac{5}{2},\frac{7}{2},4\right)$	3
Equally Important (EI)	(1,1,1,1)	1
Intermediate Scale	$\left(x-1,x-\frac{1}{2},x+\frac{1}{2},x+1\right)$	x=2,4,6,8

**Step-3.1: Trapezoidal Fuzzy Comparison of each Criterion with respect to Best criteria:** In this step we compare each criteria of set *C* with respect to selected best criteria $c_{B}\;\in \;C$ and this is described by Table 2 in below. Here ${\hat{x}}_{Bi}\;\;(i=1,2,...,n)$ takes any one of trapezoidal fuzzy measure of importance from Table 1 and ${\hat{x}}_{BB}=\left(1,1,1,1\right)$.

**Table 2:** Pairwise comparison between each criteria with respect to best criteria.

	$c_{1}$	$c_{2}$	. . .	$c_{n}$
$c_{B}$	${\hat{x}}_{B1}$	${\hat{x}}_{B2}$	. . .	${\hat{x}}_{Bn}$

**Step-3.2: Trapezoidal Fuzzy Comparison of each Criterion with respect to Worst criteria:** In this step we compare the worst criteria $c_{w}\;\in \;C$ with respect to every criteria of set*C* and this is depicted by Table 3 in below. Here, ${\hat{x}}_{iw}\;\;(i=1,2,...,n)$ takes any one of trapezoidal fuzzy measure of importance from Table 1 and ${\hat{x}}_{ww}=\left(1,1,1,1\right)$.

**Table 3:** Pairwise comparison of worst criteria with respect to each set of criteria.

	$c_{w}$
$c_{1}$	${\hat{x}}_{1w}$
$c_{2}$	${\hat{x}}_{2w}$
. . .	. . .
$c_{n}$	${\hat{x}}_{nw}$

**Step-4: Optimal Trapezoidal Fuzzy PV:** In this step determine the optimal trapezoidal fuzzy PV. Let ${\hat{w}}_{1},{\hat{w}}_{2},...,{\hat{w}}_{n}$ be the PV of criteria $c_{1},c_{2},...,c_{n}$ respectively and ${\hat{w}}_{1},{\hat{w}}_{2},...,{\hat{w}}_{n}$ are the decision variable of the optimization techniques. Let ${\hat{w}}_{B}$ and ${\hat{w}}_{w}$ be the priority values (PVs) of the criteria $c_{B}$ and $c_{w}$ respectively.

For optimal trapezoidal fuzzy PV two ratios $\frac{{\hat{w}}_{B}}{{\hat{w}}_{i}}$ and $\frac{{\hat{w}}_{i}}{{\hat{w}}_{w}}$(i=1,2,...,n) satisfies $\frac{{\hat{w}}_{B}}{{\hat{w}}_{i}}-{\hat{x}}_{Bi}=0$ and $\frac{{\hat{w}}_{i}}{{\hat{w}}_{w}}-{\hat{x}}_{iw}=0$, ∀i=1,2,...,n and it is possible for all i, if it should evaluate the minimum of maximum gaps of $\left|\frac{{\hat{w}}_{B}}{{\hat{w}}_{i}}-{\hat{x}}_{Bi}\right|$ and $\left|\frac{{\hat{w}}_{i}}{{\hat{w}}_{w}}-{\hat{x}}_{iw}\right|$, ∀i=1,2,...,n. Here all this PV in TrFBWAHP are Trapezoidal fuzzy sets (TrFS). Some time, we suggest to use ${\hat{w}}_{i}=\left(l_{{\hat{w}}_{i}},m_{{\hat{w}}_{i}},n_{{\hat{w}}_{i}},s_{{\hat{w}}_{i}}\right)$, i=1,2,...,n for optimal indicator selection. Thus the optimization problem can be written as

(1) $$\begin{array}{c} \min\;\underset{i}{\max}\left\{\left|\frac{{\hat{w}}_{B}}{{\hat{w}}_{i}}-{\hat{x}}_{Bi}\right|,\;\left|\frac{{\hat{w}}_{i}}{{\hat{w}}_{w}}-{\hat{x}}_{iw}\right|\right\}\\ Subject\;to\;\sum_{i=1}^{n}R\left({\hat{w}}_{i}\right)=1\\ l_{{\hat{w}}_{i}}\leq m_{{\hat{w}}_{i}}\leq n_{{\hat{w}}_{i}}\leq s_{{\hat{w}}_{i}},\;\forall i=1,2,\ldots ,n\;\\ l_{{\hat{w}}_{i}}\geq 0,\;\forall i=1,2,\ldots ,n \end{array}\}$$

where ${\hat{w}}_{B}=\left(l_{{\hat{w}}_{B}},m_{{\hat{w}}_{B}},n_{{\hat{w}}_{B}},s_{{\hat{w}}_{B}}\right)$, ${\hat{w}}_{i}=\left(l_{{\hat{w}}_{i}},m_{{\hat{w}}_{i}},n_{{\hat{w}}_{i}},s_{{\hat{w}}_{i}}\right)$, ${\hat{w}}_{w}=\left(l_{{\hat{w}}_{w}},m_{{\hat{w}}_{w}},n_{{\hat{w}}_{w}},s_{{\hat{w}}_{w}}\right)$, ${\hat{x}}_{Bi}=\left(l_{{\hat{x}}_{Bi}},m_{{\hat{x}}_{Bi}},n_{{\hat{x}}_{Bi}},s_{{\hat{x}}_{Bi}}\right)$ and ${\hat{x}}_{Wi}=\left(l_{{\hat{x}}_{Wi}},m_{{\hat{x}}_{Wi}},n_{{\hat{x}}_{Wi}},s_{{\hat{x}}_{Wi}}\right)$.

Next we convert the optimization problem (1) into a non-linear optimization. The transformed nonlinear form of the Eq. (1) is of the form

(2) $$\begin{array}{c} \min\;\;\hat{\zeta }\\ Subject\;to\left|\frac{{\hat{w}}_{B}}{{\hat{w}}_{i}}-{\hat{x}}_{Bi}\right|\leq \hat{\zeta }\\ \left|\frac{{\hat{w}}_{i}}{{\hat{w}}_{w}}-{\hat{x}}_{iw}\right|\leq \hat{\zeta }\\ \sum_{i=1}^{n}R\left({\hat{w}}_{i}\right)=1\\ l_{{\hat{w}}_{i}}\leq m_{{\hat{w}}_{i}}\leq n_{{\hat{w}}_{i}}\leq s_{{\hat{w}}_{i}},\;\forall i=1,2,\ldots ,n\;\\ l_{{\hat{w}}_{i}}\geq 0,\;\forall i=1,2,\ldots ,n \end{array}\}$$

where, $\hat{\zeta }=\left(l_{\hat{\zeta }},m_{\hat{\zeta }},n_{\hat{\zeta }},s_{\hat{\zeta }}\right)$

where $\hat{\zeta }$ is a trapezoidal fuzzy number thus $l_{\hat{\zeta }}\leq m_{\hat{\zeta }}\leq n_{\hat{\zeta }}\leq s_{\hat{\zeta }}$.

Now we consider the equality condition among all the components of the trapezoidal fuzzy number to minimize the value of the objective function. This equal value is defined as λ(≥0), where $\lambda =l_{\hat{\zeta }}=m_{\hat{\zeta }}=n_{\hat{\zeta }}=s_{\hat{\zeta }}$

Therefore $\hat{\zeta }=\left(\lambda ,\lambda ,\lambda ,\lambda \right)$. Thus the transformed equation of (2) is

(3) $$\begin{array}{c} \;\;\;\;\;\;\;\;\;\;\;\;\;\;\;\;\;\;\;\;\;\;\;\;\;\;\;\;\;\;\;\;\;\;\min\;\;\left(\lambda ,\lambda ,\lambda ,,\lambda \right)\\ Subject\;to\left|\frac{\left(l_{{\hat{w}}_{B}},m_{{\hat{w}}_{B}},n_{{\hat{w}}_{B}},s_{{\hat{w}}_{B}}\right)}{\left(\mu_{{\hat{w}}_{i}},\nu_{{\hat{w}}_{i}},\pi_{{\hat{w}}_{i}}\right)}-\left(l_{{\hat{x}}_{Bi}},m_{{\hat{x}}_{Bi}},n_{{\hat{x}}_{Bi}},s_{{\hat{x}}_{Bi}}\right)\right|\leq \left(\lambda ,\lambda ,\lambda ,\lambda \right)\\ \;\;\;\;\;\;\;\;\;\;\;\;\;\;\;\;\;\;\;\;\;\left|\frac{\left(l_{{\hat{w}}_{i}},m_{{\hat{w}}_{i}},n_{{\hat{w}}_{i}},s_{{\hat{w}}_{i}}\right)}{\left(l_{{\hat{w}}_{w}},m_{{\hat{w}}_{w}},n_{{\hat{w}}_{w}},,s_{{\hat{w}}_{w}}\right)}-\left(l_{{\hat{x}}_{Wi}},m_{{\hat{x}}_{Wi}},n_{{\hat{x}}_{Wi}},s_{{\hat{x}}_{Wi}}\right)\right|\leq \left(\lambda ,\lambda ,\lambda ,\lambda \right)\\ \;\;\;\;\;\;\;\;\;\;\;\;\;\;\;\;\;\;\;\;\;\sum_{i=1}^{n}R\left({\hat{w}}_{i}\right)=1\\ \;\;\;\;\;\;\;\;\;\;\;\;\;\;\;\;\;\;\;\;\;\;l_{{\hat{w}}_{i}}\leq m_{{\hat{w}}_{i}}\leq n_{{\hat{w}}_{i}}\leq s_{{\hat{w}}_{i}},\;\forall i=1,2,...,n\;\\ \;\;\;\;\;\;\;\;\;\;\;\;\;\;\;\;\;\;\;\;\;\;l_{{\hat{w}}_{i}}\geq 0,\;\forall i=1,2,...,n \end{array}\}$$

Solving the Eq. (3) we get the optimal trapezoidal fuzzy PV $\left({{\hat{w}}_{1}}^{*},{{\hat{w}}_{2}}^{*},...,{{\hat{w}}_{n}}^{*}\right)$ where, ${{\hat{w}}_{i}}^{*}=\left(l_{{{\hat{w}}_{i}}^{*}},m_{{{\hat{w}}_{i}}^{*}},n_{{{\hat{w}}_{i}}^{*}},s_{{{\hat{w}}_{i}}^{*}}\right),\forall \;i=1,2,...,n$ of the criteria.

**Step-5: Check Consistency ratio for Trapezoidal fuzzy BWM (TrFBWM):** Consistency ratio (CR) is the most significant value to examine the degree of consistency in a pairwise comparison. In Trapezoidal fuzzy BWM, a pairwise comparison is consistent matrix if ${\hat{x}}_{Bi}\times {\hat{x}}_{iW}-{\hat{x}}_{BW}=0$. If ${\hat{x}}_{Bi}\times {\hat{x}}_{iW}-{\hat{x}}_{BW}>\;or\;<0$ then the pairwise comparison matrix is inconsistent. When ${\hat{x}}_{BW}$ is equal to both ${\hat{x}}_{Bi}$ and ${\hat{x}}_{iW}$ then the inequality will reach the greatest, which output in $\hat{\zeta }$.Here we consider the existence of inequality, as per the relation$\left(\frac{{\hat{w}}_{B}}{{\hat{w}}_{i}}\right)\times \left(\frac{{\hat{w}}_{i}}{{\hat{w}}_{w}}\right)-\left(\frac{{\hat{w}}_{B}}{{\hat{w}}_{w}}\right)=0$, we can obtain the following Eq. (4) as

(4) $$\left({\hat{x}}_{Bi}-\hat{\zeta }\right)\times \left({\hat{x}}_{iW}-\hat{\zeta }\right)-\left({\hat{x}}_{BW}-\hat{\zeta }\right)=0$$

For the maximum trapezoidal fuzzy inconsistency ${\hat{x}}_{Bi}={\hat{x}}_{iW}={\hat{x}}_{BW}$, thus the equation transformed into

(5) $${\hat{\zeta }}^{2}-\left(1+2{\hat{x}}_{BW}\right)\hat{\zeta }+\left({{\hat{x}}^{2}}_{BW}-{\hat{x}}_{BW}\right)=0$$

where, $\hat{\zeta }=\left(l_{\hat{\zeta }},m_{\hat{\zeta }},n_{\hat{\zeta }},s_{\hat{\zeta }}\right)$ is a TrFS and ${\hat{x}}_{BW}=\left(l_{BW},m_{BW},n_{BW},s_{BW}\right)$

The maximum possible trapezoidal fuzzy value ${\hat{x}}_{BW}=\left(l_{BW},m_{BW},n_{BW},s_{BW}\right)$ is $\left(8,\frac{17}{2},9,9\right)$, thus $l_{BW}=8,m_{BW}=\frac{17}{2},n_{BW}=9\;and\;s_{BW}=9$. It is clear that $\max\left\{l_{BW},m_{BW},n_{BW},s_{BW}\right\}$ cannot exceed the value 9, thus the consistency index (CI) for TrFS is 13.77 (using Eq. (5)). Similarly using the same process we get the CI for TrFS. Table 4 shows CI value of TrFS.

**Table 4:** Consistency Index of TrFS.

(l,m,n,s)	Consistency Index (CI)
$\left(8,\frac{17}{2},9,9\right)$	13.77
$\left(6,\frac{13}{2},\frac{15}{2},8\right)$	12.58
$\left(4,\frac{9}{2},\frac{11}{2},6\right)$	10
$\left(2,\frac{5}{2},\frac{7}{2},4\right)$	7.37
(1,1,1,1)	3

**Step-6: Crisp PV:** In the final step, we convert optimal trapezoidal fuzzy PV $\left({{\hat{w}}_{1}}^{*},{{\hat{w}}_{2}}^{*},...,{{\hat{w}}_{n}}^{*}\right)$ where, ${{\hat{w}}_{i}}^{*}=\left(l_{{{\hat{w}}_{i}}^{*}},m_{{{\hat{w}}_{i}}^{*}},n_{{{\hat{w}}_{i}}^{*}},s_{{{\hat{w}}_{i}}^{*}}\right),\forall \;i=1,2,...,n$ into crisp value using the formula (6).

(6) $$R({{\hat{w}}_{i}}^{*})=\frac{l_{{{\hat{w}}_{i}}^{*}}+2m_{{{\hat{w}}_{i}}^{*}}+2n_{{{\hat{w}}_{i}}^{*}}+s_{{{\hat{w}}_{i}}^{*}}}{6},\;\forall \;i=1,2,...,n$$

**Step-7: Normalized PVs:** Normalized PVs of criteria are calculated by using Eq. (7).

(7) $${{\hat{w}}_{i}}^{*}=\frac{R({{\hat{w}}_{i}}^{*})}{\sum_{i=1}^{n}R({{\hat{w}}_{i}}^{*})},\;\forall i=1,2,...,n$$

**Phase-2:** After determining the PV of each criterion (by TrFBWM), Trapezoidal Fuzzy Analytic Hierarchy Process (TrFAHP) is used to find the local PV of each indicator with respect to each criterion. The proposed TrFAHP techniques consist of five steps. The evaluation steps are shown as follows:

**Step-1: Trapezoidal fuzzy judgement matrices:** In step 1 build trapezoidal fuzzy pairwise comparison matrix of indicators with respect to each criterion $C_{k}\left(k=1,2,...,n\right)$ is $A_{k}={\left[{\hat{f}}_{ij}\left(k\right)\right]}_{p\times p}$ where ${\hat{f}}_{ij}\left(k\right)=\left(l_{ij}\left(k\right),m_{ij}\left(k\right),n_{ij}\left(k\right),s_{ij}\left(k\right)\right)$ is trapezoidal fuzzy measures of importance of *i*th indicator with *j*th indicator with help of Table 1 for *k*th criteria. For checking consistency of each trapezoidal fuzzy judgement matrices, we convert each trapezoidal fuzzy judgement into score indices. The SI has calculated by the Eq. (6). We convert trapezoidal fuzzy judgement into score indices and then the pairwise comparison matrix will be transform into as normal crisp pairwise comparison matrix of AHP. So consistency ratio (CR) of pairwise comparison matrix calculates normal AHP process. If CR < 1.0 of transform trapezoidal fuzzy judgement matrices by SI then there corresponding trapezoidal fuzzy judgement matrices is consistent otherwise inconsistent.

**Step-2: Local PVs:** Now calculate the local PVs of indicators with the help of the Eq. (8).

$$a_{j}\left(k\right)={\left[\prod_{j=1}^{p}l_{ij}\left(k\right)\right]}^{\frac{1}{p}},\;\forall j=1,2,...,p$$

$$b_{j}\left(k\right)={\left[\prod_{j=1}^{p}m_{ij}\left(k\right)\right]}^{\frac{1}{p}},\;\forall j=1,2,...,p$$

$$d_{j}\left(k\right)={\left[\prod_{j=1}^{p}n_{ij}\left(k\right)\right]}^{\frac{1}{p}},\;\forall j=1,2,...,p$$

$$e_{j}\left(k\right)={\left[\prod_{j=1}^{p}s_{ij}\left(k\right)\right]}^{\frac{1}{p}},\;\forall j=1,2,...,p$$

And

$$a\left(k\right)=\sum_{j=1}^{p}a_{j}\left(k\right)$$

$$b\left(k\right)=\sum_{j=1}^{p}b_{j}\left(k\right)$$

$$d\left(k\right)=\sum_{j=1}^{p}d_{j}\left(k\right)$$

$$e\left(k\right)=\sum_{j=1}^{p}e_{j}\left(k\right)$$

Then the triangular fuzzy PV of *j*th indicator with respect to *k*th criteria is defined as follows:

(8) $${\hat{w}}_{A_{j}}\left(k\right)=\left(a_{j}^{-1}e,b_{j}^{-1}d,d_{j}^{-1}b,e_{j}^{-1}a\right),\;\;\forall j=1,2,\ldots ,p$$

In this way find the PVs of each indicator $A_{j}\;\left(j=1,2,...,p\right)$ as per each criterion $C_{k}\;\left(k=1,2,...,n\right)$.

**Step-3: Crisp PV:** Using Eq. (6), converts each fuzzy PV of each indicator $A_{j}\;\left(j=1,2,...,p\right)$ into crisp PV.

**Step-4: Normalized PVs:** Using Eq. (7), converts each crisp PV of each indicator $A_{j}\;\left(j=1,2,...,p\right)$ into normalized PV.

**Step-5: Global PVs:** Global indicators PVs are calculated by multiplying the local PV of the indicator with local PV of criteria.

**Step-6: Normalized Global PVs:** Finally global PVs convert into normalized form using Eq. (7).

## Methodology

The objective is studying a hybrid MCDM method namely TrFBWAHP based on trapezoidal fuzzy sets. This novel approach is used on the identification of the most significant indicators of efficiency of any HPP under certain uncertainties like climate change. Currently, production of power from hydropower plants is affected because of the consequence of climatic change. The hydrology is exaggerated which sequentially changes the performance efficiency of hydropower plants. Changes in the climatic pattern are the result of boost in atmospheric concentrations of greenhouse gases occurring from human activities. A lot of greenhouse gases, such as carbon dioxide ($CO_{2}$), present in the nature and maintain hotness of the earth, locking the heat in the atmosphere. Enlarged reservoir evaporation (Allawi et al., 2020), amount change (Hidalgo et al., 2020) and changes in pattern of river runoff (Dandapat, Gnanaseelan & Parekh, 2020) will have numerous impacts on the hydroelectric power production due to changes in the climate. These cause bad effects on other energy sectors (Akram et al., 2020), financial effects (Ma, Rogers & Zhou, 2020) and system operation (Bento, 2020).

Climate change is one of the great challenges of the 21st century. Hydropower is the central renewable and small carbon energy source generating 15% of total world electrical energy. On a global scale, the effect of climate change on hydropower energy production will have an impact, and this impact will be strong differences between the dry and wet regions. In areas where hydropower generation will decrease due to climate change impacts, entire nations may find themselves without a reliable source of electricity, which could be compensated by new power plants, an increase of their efficiency, and better water management.

Performance efficiency of a hydro power plant is defined as the efficiency with which the hydro power plant produces the power converting the mechanical energy of the water of the river flowing on the turbine. Hydropower energy is extensively utilized all over the world. This is the one renewable energy, currently used on the huge amount. For keeping the hydropower plant in excellent state, the power plant performance requires to be examined continuously (Zulkifli et al., 2015). Briongos et al. (2020) calculated the operating efficiency of a hybrid wind–hydro power plant situated in El Hierro Island. A new technique is presented to determine the round trip energy efficiency of hydropower plants connected to pumped storage along with underground lower reservoir (Menéndez et al., 2020).

Recently MCDM finds its huge applications in various decision making problems of renewable energy (Kumar et al., 2017). Majumder, Majumder & Saha (2018) utilized harmonic mean hierarchy process (HMHP) and measuring attractiveness by a categorical-based evaluation technique (MACBETH) methods as multi criteria decision-making techniques and polynomial neural networks to forecast the function which will represent the present position of the power plant. Majumder & Saha (2019) suggested a hybridized model using Decision Making Trial and Evaluation Laboratory (DEMATEL) with the Analytic Hiera-rchy Process (AHP) for estimating the plant efficiency of hydro power plant. Various types of multi-criteria decision making methods namely Fuzzy analytic hierarchy process, Fuzzy weighted sum model, Fuzzy analytic network process were used in detection of the significant indicator from a group of indicators for analyzing the performance reliability in hydropower plants (Majumder et al., 2019).

According to the literature review, it was found that the factors, Storage capacity (A_1_) (Majumder, 2016), Turbine efficiency (A_2_) (Hammid, Sulaiman & Abdalla, 2018), Rate of flow or discharge (A_3_) (Majumder, 2016; Majumder & Saha, 2016), Capacity factor (A_4_) (Sözen, Alp & Kilinc, 2012), Utilization factor (A_5_) (Dinkar & Morankar, 2015), Hydraulic head (A_6_) (Majumder, 2016; Baede et al., 2001) of efficiency of HPP are impacted with some climate parameters like Temperature (C_1_) (Scavia et al., 2002; Farmer, 2015), Precipitation (C_2_) (Trenberth, 2014; Cubasch, Voss & Mikolajewicz, 2000) and Evapo-transpiration (C_3_) (Abtew & Melesse, 2013). Now we discuss about all the criteria and indicators.

**Storage capacity:** The HPPs with impoundments like reservoir or dams provide the facility of storage of water that can be used for electricity generation. Due to precipitation watershed runoff carries and deposit sediments of different size into the reservoir which reduces the storage capacity of the reservoir. Again increase in temperature or drought like conditions will reduce the water level and increase the salinity of the stored resource which may cause corrosion in the turbines and thereby the HPP will be enforced to work at below capacity level. That is why, more the storage capacity less will be the vulnerability and vice-versa (Majumder, 2016).

**Turbine efficiency:** The turbine being the core component of hydropower systems performs the function of converting the hydraulic energy into electrical energy with the help of mechanical energy (Hammid, Sulaiman & Abdalla, 2018). Thus the mechanical efficiency largely influences the efficiency of hydro power plants. That is why even if there is a large flow rate available in the river due to reduced efficiency of turbines HPP will operate below capacity level. Excess precipitation carries sediments of different sizes from the catchment. The quality of water also get effected due to the flushed out pesticides and fertilizers. The suspended sediments of specific size can damage the turbine blades. Mixing of salt in the water will increase the salinity which may corrode the blades. Both of this phenomena will decrease the turbine efficiency. That is why more the precipitation more will be vulnerability to plant production due to the probability of decreased operating efficiency of the turbines.

**Rate of flow or discharge:** The rate of flow or discharge of water to the turbine largely affects the power generation of a HPP. Change in the climatic conditions results in the variation of annual precipitation and thus regular pattern of stream flow also changes. The discharge depends upon rainfall and evapo-transpiration (Majumder, 2016). And climate change brings changes in precipitation and evapo-transpiration. High temperature may lead to high evaporation and thereby reducing the availability of water whereas increased precipitation may increase the discharge. Thus climate change also affects this parameter (Majumder, 2016; Majumder & Saha, 2016).Therefore climate change creates a direct impact on capacity of a hydro power plant. Thus vulnerability of HPP is inversely proportional to discharge of the river on which the HPP is installed.

**Capacity factor:** Capacity factor is the ratio of the energy generated with a time interval to the total power possible to be generated from HPP (Sözen, Alp & Kilinc, 2012). Climate change may result into drought and reduces the potential energy of water. Again an increased precipitation increases the water level which can be converted to produce kinetic energy in the river. A plant working at below capacity level indicates that it is working below potential and vice-versa. So, capacity factor varies inversely with vulnerability of the HPP.

**Utilization factor:** Utilization Factor can be defined as the ratio of power that was generated to the power that can be generated. This factor depicts the efficiency of the powerplant in utilizing the available power potential. More the utilization less will be the vulnerability. Rather than the climatic factors the efficiency of penstocks, turbines and generators effect the magnitude of utilization factor of a HPP (Dinkar & Morankar, 2015).

**Hydraulic head:** Hydraulic head is the measurement of liquid pressure of any static water column in terms of height of water level above any datum. The performance and electricity generation of a hydropower plant directly depends on this parameter. More the hydraulic head more will be the potential energy which can be converted to flowing water and less will be the vulnerability of HPP and vice versa (Majumder, 2016; Baede et al., 2001).

**Temperature:** The global temperature of earth is referred here which is a significant factor of climate change in the present world. “A rise in global average surface temperatures is the best-known indicator of a warming climate change” (Scavia et al., 2002; Farmer, 2015). Not only global temperature the regional temperature and also local heat islands can also change the climate of a location. Climatic impact can be best represented by this parameter as temperature is responsible for occurrence of precipitation, change in wind speed, humidity and even evapo-transppiration. Thus this parameter was included in the decision hierarchy as Secondary criteria which can represent the characteristic of the climatic abnormality.

**Precipitation:** Precipitation is a form of water dropping on the earth’s surface from the atmosphere that includes rain, snow, hail, dew, fog etc. Although it is mainly a function of temperature but the impact of precipitation in the availability of water has such a significance which merit the selection of this parameter as a Secondary criteria (Trenberth, 2014). Abrupt change in the climatic conditions is characterized by abnormal precipitation due to which water level gets altered and thereby affecting the global hydro cycle (Cubasch, Voss & Mikolajewicz, 2000). It is a parameter that effects the climate change and itself also gets modified by changing climate (Cubasch, Voss & Mikolajewicz, 2000).

**Evapo-transpiration:** Evapo-transpiration is defined as the movement of water from the earth’s surface to the atmosphere through evaporation and transpiration in plants. The magnitude of Evapo-transpiration is effected by variation in both temperature and rate of transpiration. Evapo-transpiration also effect the net water balance of a catchment. The energy available in the flowing water also depends on the net water availability in the reservoir or in the catchment. The rise of temperature and decrement in the rainfall increases the evapo-transpiration rate and reduces the water availability (Abtew & Melesse, 2013). Thus evapo-transpiration takes part in climate change and is also affected by the changing climate (Abtew & Melesse, 2013). That is why this parameter is also included in the decision hierarchy as secondary criteria.

**Figure 2:**
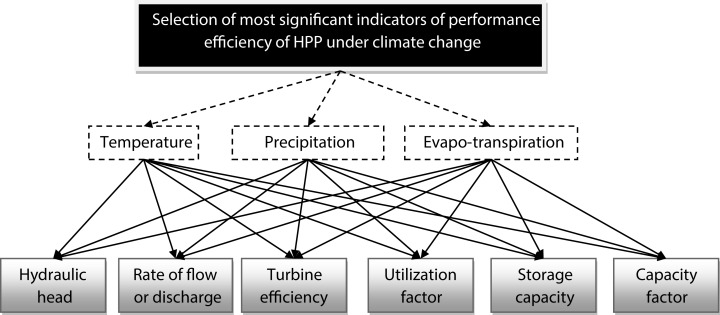
Decision Hierarchy of the current problem.

In this present study, all these factors are considered as indicators and all climate parameters are considered as criteria for applying the proposed new decision-making method. Figure 2 represent the hierarchical structure of the considering decision problem. Mathematically the objective can be written by the Eq. (9).

(9) $$e=f(A,\;W_{A})$$

where, *e* represents efficiency of HPP, $A=\left\{A_{1},A_{2},A_{3},A_{4},A_{5},A_{6}\right\}$ and *W*_*A*_ represents the set of indicator and their corresponding PV or PV of each indicator respectively.

(10) $$A_{i}=F\left(C,W_{c}\right),i=\{1,2,3,4,5,6\}$$

where, *A*_*i*_ represents indicators, $C=\left\{C_{1},C_{2},C_{3}\right\}$ and *W*_*C*_ represents the set of criteria and their corresponding PV of each criteria respectively.

**Figure 3:**
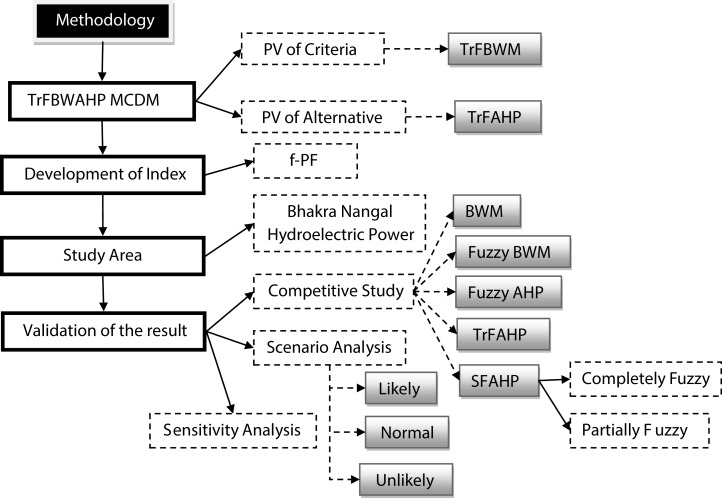
Schematic diagram of methodology.

For finding the PV of set of criteria (*W*_*C*_) and indicators (*W*_*A*_), we use new MCDM approach and computational process of the new MCDM technique discussed in “Application of TrFBWAHP MCDM”. Figure 3 shows the schematic diagram of detailed methodology.

### Application of TrFBWAHP MCDM

The objective of this section is to evaluate the PV of each criterion and indicator. So this current section divided into two parts namely “Application of TrFBWM” and “Application of TrFAHP”. In “Application of TrFBWM”, is used to determine the PV of criteria and “Application of TrFAHP” is used to evaluate the PV of each indicator with the help of criteria PV.

#### Application of TrFBWM

Here first we choose the best and worst criteria according to group of experts for the majority. After that, the pair wise comparison of each criterion is done by best criteria which are present in Table 5 in below. Similarly, another pairwise comparison is done by worst criteria with the help of each criterion which is shown by Table 6. After construction of pairwise comparison matrix we formulate the decision making optimization problem and Eq. (1) represent this mathematical formulation.

**Table 5:** Pair-wise assessment between each criterion as per best criteria.

	C_1_	C_2_	C_3_
C_1_ (Best Criteria)	EI	VSI	AMI

**Table 6:** Pairwise assessment of worst criteria as per each set of criteria.

	C_3_ (Worst Criteria)
C_1_	AMI
C_2_	ESI
C_3_	EI

(11) $$\begin{array}{c} \min\;\;\left(\lambda ,\lambda ,\lambda \right)\\ Subject\;to\left|\frac{\left(l_{{\hat{w}}_{B}},m_{{\hat{w}}_{B}},n_{{\hat{w}}_{B}},s_{{\hat{w}}_{B}}\right)}{\left(l_{{\hat{w}}_{1}},m_{{\hat{w}}_{1}},n_{{\hat{w}}_{1}},s_{{\hat{w}}_{1}}\right)}-\left(l_{{\hat{x}}_{B1}},m_{{\hat{x}}_{B1}},n_{{\hat{x}}_{B1}},s_{{\hat{x}}_{B1}}\right)\right|\leq \left(\lambda ,\lambda ,\lambda ,\lambda \right)\\ \left|\frac{\left(l_{{\hat{w}}_{B}},m_{{\hat{w}}_{B}},n_{{\hat{w}}_{B}},s_{{\hat{w}}_{B}}\right)}{\left(l_{{\hat{w}}_{2}},m_{{\hat{w}}_{2}},n_{{\hat{w}}_{2}},s_{{\hat{w}}_{2}}\right)}-\left(l_{{\hat{x}}_{B2}},m_{{\hat{x}}_{B2}},n_{{\hat{x}}_{B2}},s_{{\hat{x}}_{B2}}\right)\right|\leq \left(\lambda ,\lambda ,\lambda ,\lambda \right)\\ \;\;\;\;\;\;\;\;\;\;\;\;\;\;\;\;\;\;\;\;\;\left|\frac{\left(l_{{\hat{w}}_{B}},m_{{\hat{w}}_{B}},n_{{\hat{w}}_{B}},s_{{\hat{w}}_{B}}\right)}{\left(l_{{\hat{w}}_{3}},m_{{\hat{w}}_{3}},n_{{\hat{w}}_{3}},s_{{\hat{w}}_{3}}\right)}-\left(l_{{\hat{x}}_{B2}},m_{{\hat{x}}_{B2}},n_{{\hat{x}}_{B2}},s_{{\hat{x}}_{B2}}\right)\right|\leq \left(\lambda ,\lambda ,\lambda ,\lambda \right)\;\;\\ \;\;\;\;\;\;\;\;\;\;\;\;\;\;\;\;\;\;\;\;\;\left|\frac{\left(l_{{\hat{w}}_{1}},m_{{\hat{w}}_{1}},n_{{\hat{w}}_{1}},s_{{\hat{w}}_{1}}\right)}{\left(l_{{\hat{w}}_{w}},m_{{\hat{w}}_{w}},n_{{\hat{w}}_{w}},s_{{\hat{w}}_{w}}\right)}-\left(l_{{\hat{x}}_{W1}},m_{{\hat{x}}_{W1}},n_{{\hat{x}}_{W1}},s_{{\hat{x}}_{W1}}\right)\right|\leq \left(\lambda ,\lambda ,\lambda ,\lambda \right)\\ \;\;\;\;\;\;\;\;\;\;\;\;\;\;\;\;\;\;\;\;\;\left|\frac{\left(l_{{\hat{w}}_{2}},m_{{\hat{w}}_{2}},n_{{\hat{w}}_{2}},s_{{\hat{w}}_{2}}\right)}{\left(l_{{\hat{w}}_{w}},m_{{\hat{w}}_{w}},n_{{\hat{w}}_{w}},s_{{\hat{w}}_{w}}\right)}-\left(l_{{\hat{x}}_{W2}},m_{{\hat{x}}_{W2}},n_{{\hat{x}}_{W2}},s_{{\hat{x}}_{W2}}\right)\right|\leq \left(\lambda ,\lambda ,\lambda ,\lambda \right)\\ \;\;\;\;\;\;\;\;\;\;\;\;\;\;\;\;\;\;\;\;\;\left|\frac{\left(l_{{\hat{w}}_{3}},m_{{\hat{w}}_{3}},n_{{\hat{w}}_{3}},s_{{\hat{w}}_{3}}\right)}{\left(l_{{\hat{w}}_{w}},m_{{\hat{w}}_{w}},n_{{\hat{w}}_{w}},s_{{\hat{w}}_{w}}\right)}-\left(l_{{\hat{x}}_{W3}},m_{{\hat{x}}_{W3}},n_{{\hat{x}}_{W3}},s_{{\hat{x}}_{W3}}\right)\right|\leq \left(\lambda ,\lambda ,\lambda ,\lambda \right)\\ \;\;\;\;\;\;\;\;\;\;\;\;\;\;\;\;\;\;\;\;\;\sum_{i=1}^{3}R\left({\hat{w}}_{i}\right)=1\\ \;\;\;\;\;\;\;\;\;\;\;\;\;\;\;\;\;\;\;\;\;\;0\leq l_{{\hat{w}}_{i}}\leq m_{{\hat{w}}_{i}}\leq n_{{\hat{w}}_{i}}\leq s_{{\hat{w}}_{i}}\leq 1,\;\;\;i=1,2,3\\ \lambda \;\geq 0 \end{array}\}$$

The Eq. (11) constructs a non-linear optimization problem. The Eq. (12) represents that non-linear optimization problem. In the next section “Application of TrFAHP”, we consider application of TrFAHP.

(12) $$\begin{array}{c} \min\;\lambda \;\\ Subject\;to\;l_{{\hat{w}}_{B}}-8\;s_{{\hat{w}}_{2}}\leq \lambda s_{{\hat{w}}_{2}},\;l_{{\hat{w}}_{B}}-8\;s_{{\hat{w}}_{2}}\geq -\lambda s_{{\hat{w}}_{2}}\\ m_{{\hat{w}}_{B}}-\frac{15}{2}\;n_{{\hat{w}}_{2}}\leq \lambda n_{{\hat{w}}_{2}},\;m_{{\hat{w}}_{B}}-\frac{15}{2}\;n_{{\hat{w}}_{2}}\geq -\lambda n_{{\hat{w}}_{2}}\\ n_{{\hat{w}}_{B}}-\frac{13}{2}\;m_{{\hat{w}}_{2}}\leq \lambda m_{{\hat{w}}_{2}},\;n_{{\hat{w}}_{B}}-\frac{13}{2}m_{{\hat{w}}_{2}}\geq -\lambda m_{{\hat{w}}_{2}}\\ s_{{\hat{w}}_{B}}-6l_{{\hat{w}}_{2}}\leq \lambda l_{{\hat{w}}_{2}},\;s_{{\hat{w}}_{B}}-6l_{{\hat{w}}_{2}}\geq -\lambda l_{{\hat{w}}_{2}}\\ l_{{\hat{w}}_{B}}-9\;s_{{\hat{w}}_{3}}\leq \lambda s_{{\hat{w}}_{3}},\;l_{{\hat{w}}_{B}}-9\;s_{{\hat{w}}_{3}}\geq -\lambda s_{{\hat{w}}_{3}}\\ m_{{\hat{w}}_{B}}-\;9n_{{\hat{w}}_{3}}\leq \lambda n_{{\hat{w}}_{3}},\;m_{{\hat{w}}_{B}}-9\;n_{{\hat{w}}_{3}}\geq -\lambda n_{{\hat{w}}_{3}}\\ n_{{\hat{w}}_{B}}-\frac{17}{2}\;m_{{\hat{w}}_{3}}\leq \lambda m_{{\hat{w}}_{3}},\;n_{{\hat{w}}_{B}}-\frac{17}{2}\;m_{{\hat{w}}_{3}}\geq -\lambda m_{{\hat{w}}_{3}}\\ s_{{\hat{w}}_{B}}-8\;l_{{\hat{w}}_{3}}\leq \lambda l_{{\hat{w}}_{3}},\;s_{{\hat{w}}_{B}}-8\;l_{{\hat{w}}_{3}}\geq -\lambda l_{{\hat{w}}_{3}}\\ l_{{\hat{w}}_{2}}-6\;s_{{\hat{w}}_{w}}\leq \lambda s_{{\hat{w}}_{w}},\;l_{{\hat{w}}_{2}}-6\;s_{{\hat{w}}_{w}}\geq -\lambda s_{{\hat{w}}_{w}}\\ m_{{\hat{w}}_{2}}-\frac{11}{2}n_{{\hat{w}}_{w}}\leq \lambda n_{{\hat{w}}_{w}},\;m_{{\hat{w}}_{2}}-\frac{11}{2}n_{{\hat{w}}_{w}}\geq -\lambda n_{{\hat{w}}_{w}}\\ n_{{\hat{w}}_{2}}-\frac{9}{2}\;m_{{\hat{w}}_{w}}\leq \lambda m_{{\hat{w}}_{w}},\;\;n_{{\hat{w}}_{2}}-\frac{9}{2}m_{{\hat{w}}_{w}}\geq -\lambda m_{{\hat{w}}_{w}}\\ s_{{\hat{w}}_{2}}-4\;l_{{\hat{w}}_{w}}\leq \lambda l_{{\hat{w}}_{w}},\;\;s_{{\hat{w}}_{2}}-4l_{{\hat{w}}_{w}}\geq -\lambda l_{{\hat{w}}_{w}}\\ \sum_{i=1}^{3}\left(\frac{l_{{\hat{w}}_{i}}+2m_{{\hat{w}}_{i}}+2n_{{\hat{w}}_{i}}+s_{{\hat{w}}_{i}}}{6}\right)=1\\ 0\leq l_{{\hat{w}}_{i}}\leq m_{{\hat{w}}_{i}}\leq n_{{\hat{w}}_{i}}\leq s_{{\hat{w}}_{i}}\leq 1,\;\;\;i=1,2,3\\ \lambda \;\geq 0 \end{array}\}$$

#### Application of TrFAHP

In the present study, **TrFAHP** has used to find the local weight of each indicator as per each criterion. Thus pairwise comparison is required for indicators as per each criterion. Tables 7–9 represents the pairwise comparison of indicators as per the criteria C_1_, C_2_ and C_3_ respectively.

**Table 7:** Pairwise comparison table of indicators caused by C_1_.

	A_1_	A_2_	A_3_	A_4_	A_5_	A_6_
A_1_	(1,1,1,1)	$\left(2,\frac{5}{2},\frac{7}{2},4\right)$	$\left(4,\frac{9}{2},\frac{11}{2},6\right)$	$\left(4,\frac{9}{2},\frac{11}{2},6\right)$	$\left(2,\frac{5}{2},\frac{7}{2},4\right)$	$\left(6,\frac{13}{2},\frac{15}{2},8\right)$
A_2_	$\left(\frac{1}{4},\frac{2}{7},\frac{2}{5},\frac{1}{2}\right)$	(1,1,1,1)	$\left(4,\frac{9}{2},\frac{11}{2},6\right)$	$\left(2,\frac{5}{2},\frac{7}{2},4\right)$	$\left(2,\frac{5}{2},\frac{7}{2},4\right)$	$\left(4,\frac{9}{2},\frac{11}{2},6\right)$
A_3_	$\left(\frac{1}{6},\frac{2}{11},\frac{2}{9},\frac{1}{4}\right)$	$\left(\frac{1}{6},\frac{2}{11},\frac{2}{9},\frac{1}{4}\right)$	(1,1,1,1)	$\left(\frac{1}{4},\frac{2}{7},\frac{2}{5},\frac{1}{2}\right)$	(1,1,1,1)	$\left(2,\frac{5}{2},\frac{7}{2},4\right)$
A_4_	$\left(\frac{1}{6},\frac{2}{11},\frac{2}{9},\frac{1}{4}\right)$	$\left(\frac{1}{4},\frac{2}{7},\frac{2}{5},\frac{1}{2}\right)$	$\left(2,\frac{5}{2},\frac{7}{2},4\right)$	(1,1,1,1)	$\left(2,\frac{5}{2},\frac{7}{2},4\right)$	$\left(4,\frac{9}{2},\frac{11}{2},6\right)$
A_5_	$\left(\frac{1}{4},\frac{2}{7},\frac{2}{5},\frac{1}{2}\right)$	$\left(\frac{1}{4},\frac{2}{7},\frac{2}{5},\frac{1}{2}\right)$	(1,1,1,1)	$\left(\frac{1}{4},\frac{2}{7},\frac{2}{5},\frac{1}{2}\right)$	(1,1,1,1)	$\left(2,\frac{5}{2},\frac{7}{2},4\right)$
A_6_	$\left(\frac{1}{8},\frac{2}{15},\frac{2}{13},\frac{1}{6}\right)$	$\left(\frac{1}{6},\frac{2}{11},\frac{2}{9},\frac{1}{4}\right)$	$\left(\frac{1}{4},\frac{2}{7},\frac{2}{5},\frac{1}{2}\right)$	$\left(\frac{1}{6},\frac{2}{11},\frac{2}{9},\frac{1}{4}\right)$	$\left(\frac{1}{4},\frac{2}{7},\frac{2}{5},\frac{1}{2}\right)$	(1,1,1,1)

**Table 8:** Pairwise comparison table of indicators caused by C_2_.

	A_1_	A_2_	A_3_	A_4_	A_5_	A_6_
A_1_	(1,1,1,1)	(1,1,1,1)	$\left(4,\frac{9}{2},\frac{11}{2},6\right)$	$\left(2,\frac{5}{2},\frac{7}{2},4\right)$	$\left(8,\frac{17}{2},9,9\right)$	$\left(2,\frac{5}{2},\frac{7}{2},4\right)$
A_2_	(1,1,1,1)	(1,1,1,1)	$\left(4,\frac{9}{2},\frac{11}{2},6\right)$	$\left(6,\frac{13}{2},\frac{15}{2},8\right)$	$\left(6,\frac{13}{2},\frac{15}{2},8\right)$	$\left(4,\frac{9}{2},\frac{11}{2},6\right)$
A_3_	$\left(\frac{1}{6},\frac{2}{11},\frac{2}{9},\frac{1}{4}\right)$	$\left(\frac{1}{6},\frac{2}{11},\frac{2}{9},\frac{1}{4}\right)$	(1,1,1,1)	$\left(2,\frac{5}{2},\frac{7}{2},4\right)$	$\left(4,\frac{9}{2},\frac{11}{2},6\right)$	$\left(2,\frac{5}{2},\frac{7}{2},4\right)$
A_4_	$\left(\frac{1}{4},\frac{2}{7},\frac{2}{5},\frac{1}{2}\right)$	$\left(\frac{1}{8},\frac{2}{15},\frac{2}{13},\frac{1}{6}\right)$	$\left(\frac{1}{4},\frac{2}{7},\frac{2}{5},\frac{1}{2}\right)$	(1,1,1,1)	$\left(2,\frac{5}{2},\frac{7}{2},4\right)$	(1,1,1,1)
A_5_	$\left(\frac{1}{9},\frac{1}{9},\frac{2}{17},\frac{1}{8}\right)$	$\left(\frac{1}{8},\frac{2}{15},\frac{2}{13},\frac{1}{6}\right)$	$\left(\frac{1}{6},\frac{2}{11},\frac{2}{9},\frac{1}{4}\right)$	$\left(\frac{1}{4},\frac{2}{7},\frac{2}{5},\frac{1}{2}\right)$	(1,1,1,1)	$\left(\frac{1}{4},\frac{2}{7},\frac{2}{5},\frac{1}{2}\right)$
A_6_	$\left(\frac{1}{4},\frac{2}{7},\frac{2}{5},\frac{1}{2}\right)$	$\left(\frac{1}{6},\frac{2}{11},\frac{2}{9},\frac{1}{4}\right)$	$\left(\frac{1}{4},\frac{2}{7},\frac{2}{5},\frac{1}{2}\right)$	(1,1,1,1)	$\left(2,\frac{5}{2},\frac{7}{2},4\right)$	(1,1,1,1)

**Table 9:** Pairwise comparison table of indicators caused by C_3_.

	A_1_	A_2_	A_3_	A_4_	A_5_	A_6_
A_1_	(1,1,1,1)	$\left(2,\frac{5}{2},\frac{7}{2},4\right)$	$\left(6,\frac{13}{2},\frac{15}{2},8\right)$	$\left(4,\frac{9}{2},\frac{11}{2},6\right)$	$\left(8,\frac{17}{2},9,9\right)$	$\left(6,\frac{13}{2},\frac{15}{2},8\right)$
A_2_	$\left(\frac{1}{4},\frac{2}{7},\frac{2}{5},\frac{1}{2}\right)$	(1,1,1,1)	$\left(2,\frac{5}{2},\frac{7}{2},4\right)$	$\left(2,\frac{5}{2},\frac{7}{2},4\right)$	$\left(6,\frac{13}{2},\frac{15}{2},8\right)$	$\left(4,\frac{9}{2},\frac{11}{2},6\right)$
A_3_	$\left(\frac{1}{8},\frac{2}{15},\frac{2}{13},\frac{1}{6}\right)$	$\left(\frac{1}{4},\frac{2}{7},\frac{2}{5},\frac{1}{2}\right)$	(1,1,1,1)	$\left(\frac{1}{4},\frac{2}{7},\frac{2}{5},\frac{1}{2}\right)$	$\left(4,\frac{9}{2},\frac{11}{2},6\right)$	$\left(\frac{1}{4},\frac{2}{7},\frac{2}{5},\frac{1}{2}\right)$
A_4_	$\left(\frac{1}{6},\frac{2}{11},\frac{2}{9},\frac{1}{4}\right)$	$\left(\frac{1}{4},\frac{2}{7},\frac{2}{5},\frac{1}{2}\right)$	$\left(2,\frac{5}{2},\frac{7}{2},4\right)$	(1,1,1,1)	$\left(6,\frac{13}{2},\frac{15}{2},8\right)$	$\left(2,\frac{5}{2},\frac{7}{2},4\right)$
A_5_	$\left(\frac{1}{9},\frac{1}{9},\frac{2}{17},\frac{1}{8}\right)$	$\left(\frac{1}{8},\frac{2}{15},\frac{2}{13},\frac{1}{6}\right)$	$\left(\frac{1}{6},\frac{2}{11},\frac{2}{9},\frac{1}{4}\right)$	$\left(\frac{1}{8},\frac{2}{15},\frac{2}{13},\frac{1}{6}\right)$	(1,1,1,1)	$\left(\frac{1}{6},\frac{2}{11},\frac{2}{9},\frac{1}{4}\right)$
A_6_	$\left(\frac{1}{8},\frac{2}{15},\frac{2}{13},\frac{1}{6}\right)$	$\left(\frac{1}{6},\frac{2}{11},\frac{2}{9},\frac{1}{4}\right)$	$\left(2,\frac{5}{2},\frac{7}{2},4\right)$	$\left(\frac{1}{4},\frac{2}{7},\frac{2}{5},\frac{1}{2}\right)$	$\left(4,\frac{9}{2},\frac{11}{2},6\right)$	(1,1,1,1)

### Development of index

Determine the PV of each indicator, an index is developed by the magnitude value and corresponding PV of each indicator. The index is taken as PV average of each magnitude value of indicators which is directly related to performance efficiency (PE) of an HPP. The function of trigonometric sine of this proportion is taken as an index. In the present study, we are assuming that just normalized quantities for the factors were to be applauded. The function Sine gives the comparison with a standardized restricted domain of index values with other accessible indicators (Choudhury et al., 2019). The bigger the value of the factor, the greater its appropriateness is considered to be. Thus, the value of the *f-PF*-mapping from (13) was taken a real number, also *f-PF*-mapping was taken to be the expansion of sine function in Taylor series where higher-order terms are ignored (Choudhury et al., 2019). The mathematical expression of the proposed index (*f-PE*) is represented by the Eq. (13).

(13) $$\;\;f-PE=\left|\sin\left(\frac{\sum_{i=1}^{6}w_{i}A_{i}}{\sum_{i=1}^{6}w_{i}}\right)\right|$$

where and *A*_*i*_ represents *i*th indicator and *w*_*i*_ represents the PV of the *i*th indicator.

### Case study

One case study is selected to evaluate the PF in different scenarios for validation of the results proposed new MCDM method. The Bhakra-Nangal Dam is second tallest dam in Asia and situated in the border of Punjab and Himachal Pradesh. It is the highest straight gravity dam in India with the height of about 207.26 m and it runs across 168.35 km. Bhakra Nangal Dam has a length of 518.25 (1,700 ft) m and a width of 9.1 m (30 ft) approximately. The Bhakra-Nangal dam is one of the earliest river valley development schemes undertaken after the independence of India. The Bhakra dam is built on Sutlej River. The dam provides irrigation water and electricity to Haryana, Rajasthan, Gujarat, and Himachal Pradesh. It has ten hydroelectric power generators on each side. Thus Bhakra-Nangal Dam is a vital dam among the entire dam in Asian nation. As a result we have contemplated it as case study to examine the affectedness of the hydro power plant by climate change. Table 10 shows the normalized data in normal Scenario of Bhakra Nangal Hydroelectric Power Project India (http://globalenergyobservatory.org/geoid/2330, https://bbmb.gov.in/bhakra.htm).

**Table 10:** Normalized data in normal scenario Bhakra Nangal hydroelectric power project India.

Factors	Normalized Data in Normal Scenario
Hydraulic head (in meter)	0.011014
Rate of flow or discharge (in Cumech)	0.84372
Turbine efficiency (in %)	0.008229
Utilization factor (in %)	0.007588
Storage capacity (in cubic meters)	0.119693
Capacity factor (in %)	0.009756

### Validation of proposed model

To validate the result of the proposed MCDM techniques, we have used three different sections in the present investigation. First, compare the result of the new technique with some existing MCDM tools (Comparative Study). Next, validation of the result by a sensitivity analysis for each considering indicator (Scenario Analysis) is done. Lastly, use scenario analysis with the help of *f-PF* index value in different scenarios of considering indicators (Sensitivity Analysis).

#### Comparative study

A comparative study is utilized to decide and measure connections between at least two factors by noticing various gatherings that either by decision or conditions is presented to various treatments. Relative investigation takes a gander at at least two comparative groups, individuals, or conditions by comparing them. In the present study, the result of the proposed method compared with some advanced techniques like BWM (Rezaei, 2015), Fuzzy BWM (Guo & Zhao, 2017), Fuzzy AHP (Chang, 1996), TrFAHP (Zheng et al., 2012) and SFAHP (spherical fuzzy analytic hierarchy process) (Gündoğdu & Kahraman, 2020).

#### Scenario analysis

There can be two types of scenarios for any system which is Likely and Unlikely scenario. The Likely scenarios are the situations which have the maximum chance of occurrence or a common situation encountered by the system. The Unlikely scenarios are those scenarios which are rare and have a minimum chance of occurrence. A system can experience both kinds of scenarios. In the present investigation to validate the developed method, outputs are made for both likely and unlikely scenarios. The likely scenarios were created by considering a variation of 1–15% (Majumder et al., 2019) in the input parameters and the unlikely scenarios were produced by varying the inputs within 15–80% (Majumder et al., 2019) about the mean value. The variation considered in the input variables is self-explanatory and can be easily differentiated between the likely and unlikely situations. The performance efficiency of the model will be ensured if realistic prediction can be achieved for both type of scenarios. The scenarios are made based on the data retrieved from the case study areas selected for case study analysis.

#### Sensitivity analysis

The sensitivity analysis (Hamby, 1994) is performed to ensure that the significance of the indicators as approximated in “Methodology” with the help of TrFBWAHP MCDM method is corroborated to the predicted output. The PV of the indicators will be same as the sensitivity of the indicators which are used as the input variable of the *f-PE* index. A Multiple Input Single Output (MISO) Tornado method developed by SenseIt Limited. The values of the input variables are varied within 0–1 and the output predicted from the model was noted. For each increment in the input the change is noted in the output. When the maximum change is observed in the output variable the increment in the inputs was noted and based on the change in the input variable and the change in output variable the sensitivity is calculated. An input parameter which has the minimum change compared to all other input parameters for which maximum change is observed in the output variable will be the most sensitive and the opposite will be the least sensitive variable. This sensitivity must be similar with the PV of the input variables and thus the most sensitive will have the maximum PV and vice-versa.

## Result and discussion

The object of the investigation is to find the best indicator for efficiency for the HPP impacted by climate change using new MCDM approach. In these aspects, the present study result divided into four parts namely result from MCDM, result from comparative study, result from scenario analysis, the result from sensitivity analysis and discussion which are described in “Result from MCDM”, “Comparative Study”, “Result from Sensitivity Analysis”, “Result from Scenario Analysis”.

### Result from MCDM

In the present decision-making problem consider three criteria and six indicators. Solve this decision-making problem with the help of a novel an MCDM method viz. TrFBWAHP. “Result from MCDM” is divided into three parts for evaluating the final PV of indicators. First, TrFBWM is used to evaluate the PV of criteria (Result from TrFBWM). Next TrFAHP is utilized to determine the local PV of each indicator (Result from TrFAHP). Results obtained from “Result from TrFBWM” and “Result from TrFAHP” are used to determine the global PV of each indicator (Result from TrFBWAHP).

#### Result from TrFBWM

The TrFBWM method was used to calculate the relative of importance for the criteria with the help of PVs. Solving the Eq. (12), the optimal trapezoidal fuzzy PVs of three criteria (“temperature”, “precipitation” and “evapo-transpiration”) can be evaluate, that are

${{\hat{w}}_{1}}^{*}=(0.847,0.847,0.847,0.847)$; ${{\hat{w}}_{2}}^{*}=(0.126,0.126,0.126,0.126)$; ${{\hat{w}}_{3}}^{*}=(0.015,0.015,0.015,0.087)$; λ=(0.750,0.750,0.750,0.750)

Next, we calculate the crisp PVs of the criteria temperature, precipitation and evapo-transpiration using Eq. (6) that are $R\left({{\hat{w}}_{1}}^{*}\right)=0.847$; $R\left({{\hat{w}}_{2}}^{*}\right)=0.126$; $R\left({{\hat{w}}_{3}}^{*}\right)=0.027$.

Again, we calculate normalized PVs values of these three criteria with the help of the Eq. (7), which are ${{\hat{w}}_{1}}^{*s}=0.847$; ${{\hat{w}}_{2}}^{*s}=0.126$; ${{\hat{w}}_{3}}^{*s}=0.027$.

The PVs of three criteria “temperature”, “precipitation” and “evapo-transpiration” are 0.590, 0.339 and 0.0741 respectively by applying existing BWM techniques (Rezaei, 2015). Also using fuzzy BWM on the same problem we get the PVs of the criteria “temperature”, “precipitation” and “evapo-transpiration” are 0.507, 0.350 and 0.143 respectively. It is clear that the ranking of each criterion are same in all the BWM, fuzzy BWM and Trapezoidal fuzzy BMW methods although there are some gaps among the PVs of those criteria. Because ${\hat{x}}_{Bi}={\hat{x}}_{B3}=\left(8,\frac{17}{2},9,9\right)$, the CI for this case is 13.77 so the CR = 0.750/13.77 is equal to 0.054. In the same problem we apply existing BWM (Rezaei, 2015) and fuzzy BWM (Guo & Zhao, 2017) then CR value is 0.580 and 0.056respectively. It can be seen that the CR value obtain by Trapezoidal fuzzy BMW approach is less than the CR obtain by BWM and fuzzy BWM approach, so it can be concluded that the Trapezoidal fuzzy BWM shows better comparison CR than the BWM and fuzzy BMW.

#### Result from TrFAHP

The TrFAHP (Zheng et al., 2012) method was used to determine the local PVs of indicators. In this study indicators were consider as indicators. Tables 7–9 represents the pairwise comparison matrix of indicators with respect to each criterion. The value of CR (Saaty, 1980) of that pairwise comparison matrix (Tables 7–9) was calculating corresponding pairwise comparison matrix of existing classical AHP approach from the linguistic scale (Table 1). Using the Eq. (6) and (10) we find the PVs of indicators with respect to each criterion. Table 11 represents the local PVs of indicators in Trapezoidal fuzzy PVs (using Eq. (10)), defuzzify the criteria PVs (using Eq. (6)) and CR value. According to the result it is clear that all the pair wise comparison matrix (Tables 7–9) are consistent because all the CR value (see in Table 11) corresponding to each matrix is less than 1.0 (Saaty, 1980).

**Table 11:** Local PVs of indicators.

	C_1_		C_2_		C_3_	
	Trapezoidal Local PVs	**Crisp PV**	Trapezoidal Local PVs	**Crisp PV**	Trapezoidal Local PVs	**Crisp PV**
A_1_	**(**0.025, 0.03, 0.044, 0.057)	0.038	**(**0.029, 0.033, 0.047, 0.058)	0.041	**(**0.019, 0.022, 0.031, 0.038)	0.027
A_2_	**(**0.039, 0.048, 0.075, 0.097)	0.064	**(**0.023, 0.027, 0.036, 0.042)	0.032	**(**0.034, 0.041, 0.061, 0.076)	0.052
A_3_	**(**0.142, 0.173, 0.254, 0.312)	0.218	**(**0.075, 0.089, 0.131, 0.164)	0.113	**(**0.129, 0.160, 0.238, 0.289)	0.203
A_4_	**(**0.066, 0.082, 0.128, 0.164)	0.108	**(**0.099, 0.124, 0.194, 0.248)	0.164	**(**0.057, 0.069, 0.103, 0.129)	0.089
A_5_	**(**0.113, 0.142, 0.219, 0.273)	0.184	**(**0.300, 0.369, 0.532, 0.637)	0.456	**(**0.371, 0.434, 0.583, 0.676)	0.514
A_6_	**(**0.270, 0.339, 0.510, 0.625)	0.432	**(**0.140, 0.179, 0.279, 0.351)	0.234	**(**0.095, 0.116, 0.170, 0.209)	0.146
CR	0.68 < 1		0.61 < 1		0.95 < 1	

#### Result from TrFBWAHP

**Figure 4:**
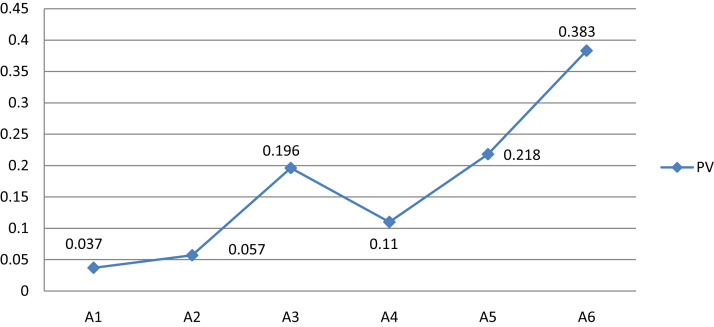
Global PVs of indicators obtained from TrFBWAHP.

The TrFBWAHP approach is applied to estimate the global PVs importance of indicators. In this stage the PV of criteria taken from TrFBWM and local PVs of indicators (or alternatives) are taken from TrFAHP. Finally global PVs convert into normalized form using Eq. (7). According to the result we can seen that both cases indicate hydraulic head is the more responsible for impact on HPP. Figure 4 shows the PV of each indicators determined by TrFBWAHP.

### Comparative study

**Figure 5:**
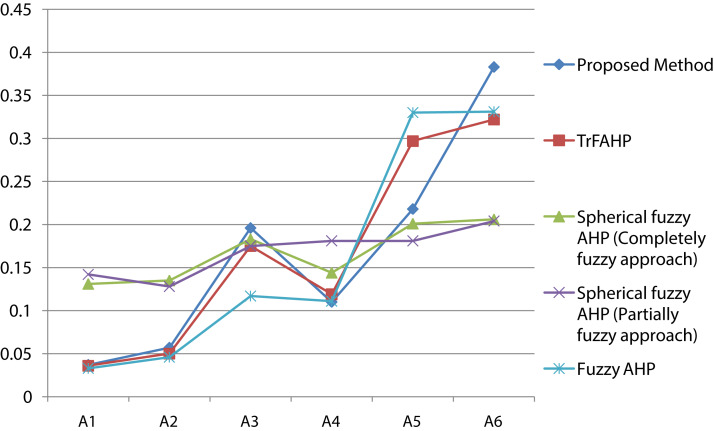
Result of comparative study.

We perform a thorough analysis with some existing methods and our proposed method in particular TrFBWAHP. Figure 5 highlights the summary about the score values and the ranking order of the given indicators. From the Fig. 5, we have observed that the proposed method exhibits the better performance as per the existing methods. If we focus on the results shown in the figure then we can understand that the best indicator of the proposed technique coincides with all other existing approaches and as results this advanced approach may be well versed due to the effectiveness of the BWM approach for criteria selection. Moreover, this figure states that although the ranking order becomes same and the optimal indicator is A_1_ that is, hydraulic head for all the approaches but the computational steps are different.

### Result from sensitivity analysis

**Figure 6:**
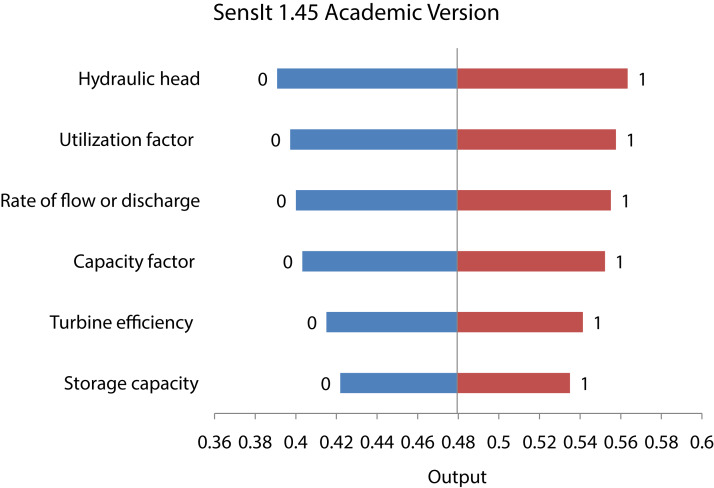
Result of sensitivity analysis.

In some studies where required a model of numerical to validate the estimated output. This type of estimation was done by Sensitivity Analysis. Figure 6 shows the result of Sensitivity Analysis. The input variables were six indicators and the output was the index function (Eq. (13)). Sensitivity of each input parameter were measured by its seing^2 value. According to the results, the Hydraulic Head (A_1_) was found to have a Swing^2 value of 22.80% whereas Utilization Factor (A_5_) was found to have a Swing^2 value of 19.7%. Thus, the Hydraulic Head (A_1_) was rated the most sensitive parameter and Utilization Factor (A_4_) the second most sensitive parameter. On the other hand, the two least sensitive parameters were found out to be Storage Capacity (A_1_) having Swing^2 value of 9.8%. It indicates that “Hydraulic Head” is the most sensitive parameter followed by vulnerability analysis of HPP with respect to the PV as determined by the TrFBWAHP.

### Result from scenario analysis

Scenario analysis was validated our proposed model. Scenario analysis consists 6 likely and 6 unlikely which was described in the sub-section “Scenario Analysis”. In Table 12 column two represents the variation of indicators to the increments depicted. The main objective of the scenario analysis was monitoring the index value in different scenario. If the model is learned between the correlation of input and output function the monitor is possible. Since in this study we consider sine of linear index so if the indicators (or decrease) are increased, then also the index value will be increased (or decrease). It can be observed that from Table 12 with respect to the current situation of the power plant likely scenario lies in 0.175664–0.176909 and unlikely scenario lies in 0.172964–0.179607. From the Table 12 it is clear that the index value is maximum under likely scenario in scenario 3 when Hydraulic Head is increase 15% and minimum in scenario 6. From the Table 12 it is clear that the index value is maximum under unlikely scenario in scenario 3 when Hydraulic Head is increase 80% and minimum in scenario 6 when Hydraulic Head is decrease 80%.

**Table 12:** Result of scenario analysis.

	Scenario	Hydraulic head (%)	Index value
Likely Scenario	Scenario 1	5	0.176494
	Scenario 2	10	0.176702
	Scenario 3	15	0.176909
	Scenario 4	−5	0.176079
	Scenario 5	−10	0.175871
	Scenario 6	−15	0.175664
Normal Scenario (*f-PF* value)			0.176286
Unlikely Scenario	Scenario 1	20	0.177117
	Scenario 2	50	0.178362
	Scenario 3	80	0.179607
	Scenario 4	−20	0.175456
	Scenario 5	−50	0.17421
	Scenario 6	−100	0.172964

## Conclusion

The main advantage of our method is to choose the trapezoidal fuzzy BWM for selecting the best and worst criteria. Further, Trapezoidal fuzzy AHP has been applied to select the best indicator. Results prove the high efficiency and good performance of the proposed method. The newly proposed hybrid method, namely TrFBWAHP is for the first time used to determine the dependable indicator for performance efficiency analysis of a hydropower plant by climate change. The results indicate that “Hydraulic head” is the most significant indicator observed by our proposed method. Some existing work also supports our result which is found by our proposed hybrid method (Perera & Rathnayake, 2019; Majumder, Majumder & Saha, 2018). Here comparative study scenario analysis and sensitivity analysis are done which also support our result. From the present work, it is to be noted that our proposed method (TrBWM) is more general with respect to existing BWM. Moreover, fuzzy BWM techniques overcome many demerits of AHP in criteria level. But in indicator level we have used existing triangular fuzzy AHP, thus the drawbacks of AHP cannot overcome from the indicator level which is a lacuna of our study. In the future, BWM will be used in the type-2 fuzzy set and neutrosophic fuzzy set.

## Supplemental Information

10.7717/peerj-cs.453/supp-1 Supplemental Information 1 Raw Data.

10.7717/peerj-cs.453/supp-2 Supplemental Information 2 MATLAB code.

## Additional Information and Declarations

### Data Availability

The following information was supplied regarding data availability: Code and data are available in the Supplemental Files.
